# Ub-POD: A Ubiquitin-Specific Proximity-Dependent Labeling Technique to Identify E3 Ubiquitin Ligase Substrates in Human Cells

**DOI:** 10.21769/BioProtoc.5351

**Published:** 2025-06-20

**Authors:** Urbi Mukhopadhyay, Sophie Levantovsky, Christian Behrends, Sagar Bhogaraju

**Affiliations:** 1 Institute of Biochemistry II, Faculty of Medicine, Goethe University Frankfurt, Frankfurt am Main, Germany; 2 European Molecular Biology Laboratory, 71 avenue des Martyrs, 38042 Grenoble, France; 3 Munich Cluster for Systems Neurology, Medical Faculty, Ludwig-Maximilians-Universität München, Munich, Germany

**Keywords:** Ub-POD, Ubiquitin, E3 ligase, Substrate identification, BirA, RING, U box, Biotinylation, Proximity labeling

## Abstract

Ubiquitination is a post-translational protein modification that regulates a vast majority of processes during protein homeostasis. The covalent attachment of ubiquitin is a highly regulated process carried out by the sequential action of the three enzymes E1, E2, and E3. E3 ligases share a dual function of 1) transferring covalently attached ubiquitin from the catalytic cysteine of E2 (E2~Ub) to the substrate and 2) providing substrate specificity. Our current knowledge of their individual substrate pools is incomplete due to the difficult capture of these transient substrate–E3 ligase interactions. Here, we present an efficient protocol that enables the selective biotinylation of substrates of a given ubiquitin ligase. In brief, the candidate E3 ligase is fused to the biotin ligase BirA and ubiquitin to a biotin acceptor peptide, an Avi-tag variant (-2) AP. Cells are co-transfected with these fusion constructs and exposed to biotin, resulting in a BirA-E3 ligase-catalyzed biotinylation of (-2) AP-Ub when in complex with E2. As the next step, the biotinylated (-2) AP-Ub is transferred covalently to the substrate lysine, which enables an enrichment via denaturing streptavidin pulldown. Substrate candidates can then be identified via mass spectrometry (MS). Our ubiquitin-specific proximity-dependent labeling (Ub-POD) method allows robust biotinylation of the ubiquitylation substrates of a candidate E3 ligase thanks to the wild-type BirA and biotin acceptor peptide fused to the E3 and Ub, respectively. Because of the highly Ub-specific labeling, Ub-POD is more appropriate for identifying ubiquitination substrates compared to other conventional proximity labeling or immunoprecipitation (IP) approaches.

Key features

• A simple and cost-effective method using common chemicals makes Ub-POD easy to implement in any laboratory.

• Can be an exploratory tool to identify new substrates via mass spectrometry (MS) or as a validation tool in combination with immunoblotting or immunofluorescence.

• Knowledge of triggers and constraints of E3 ligase activity is beneficial.

## Graphical overview


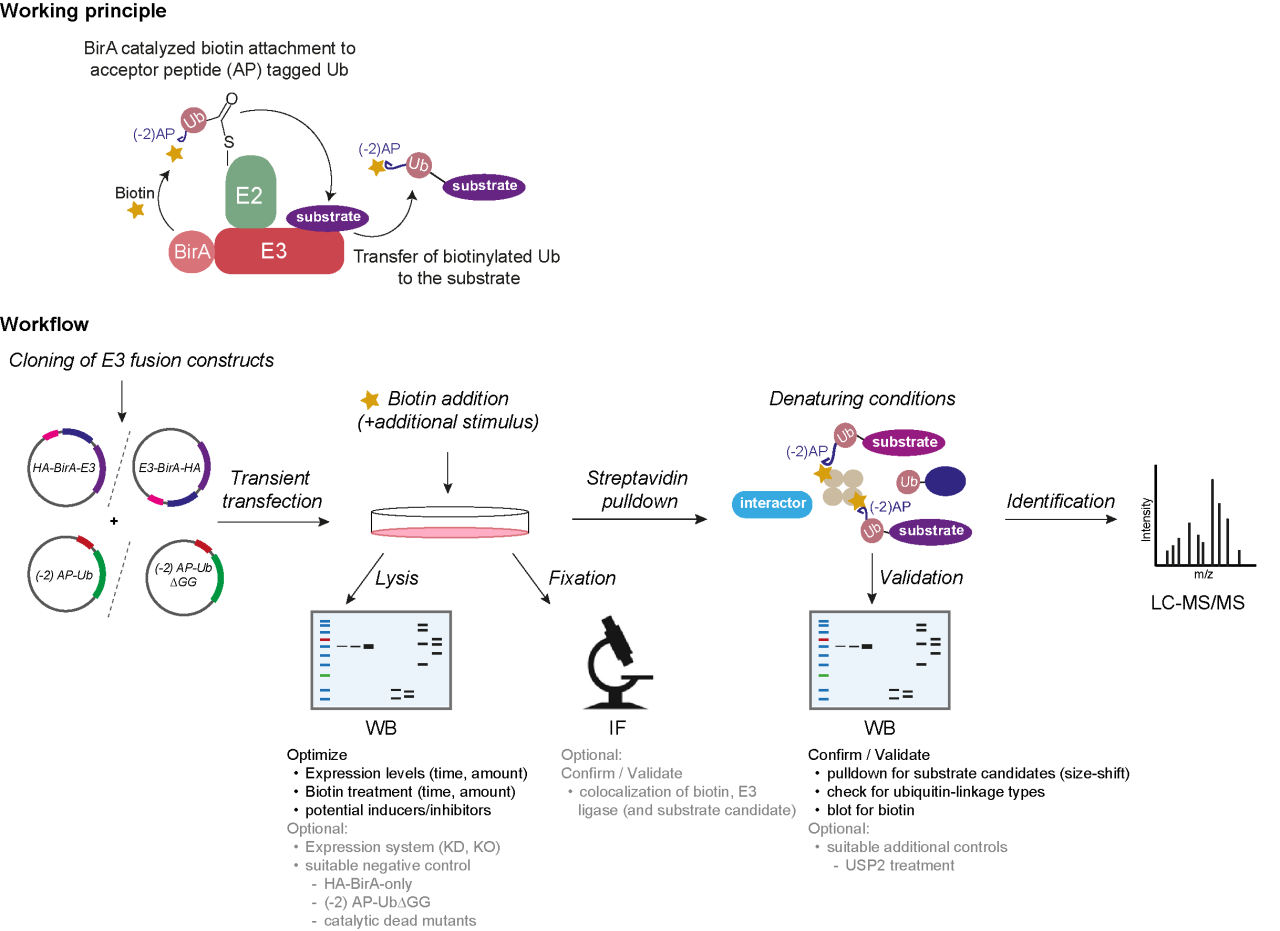


## Background

Ubiquitination is a post-translational protein modification with a pivotal role in many cellular processes, including the regulation of protein stability, enzymatic activity, cellular protein localization, and transport. Altogether, it leads to a functional conversion of proteins and regulates signaling events in a time- and spatial-dependent manner. Ubiquitin is a 76 amino acid small protein conjugated to its target by a concerted three-step enzymatic cascade, comprising the E1 activating enzyme, the E2 conjugating enzyme, and the E3 ligase [1]. The reaction is initiated with the activation of ubiquitin by one of the two E1 enzymes, which adenylates the terminal carboxyl group of ubiquitin prior to formation of a high-energy thioester bond with the catalytic cysteine of E1. Ubiquitin is then passed on to one of ~40 members of E2 via an E2-catalyzed transthioesterification reaction. Lastly, the E3 ligase interacts with the E2~Ub complex as well as with its substrate and enables the covalent attachment of the terminal carboxyl group of ubiquitin to its substrate lysine through an isopeptide bond [1–3]. Over 600 different E3 ligases are estimated to convey substrate specificity and make ubiquitination a very versatile system with over thousands of targets. E3 ligases can furthermore catalyze ubiquitination of ubiquitin itself at several lysine residues (K6, K11, K48, K63, etc.), resulting in different ubiquitin linkage chain types. The linkage type can often be interpreted as a code for different protein fates. For instance, K48 ubiquitin linkage chain types are often utilized as signals for proteasomal degradation, whereas K63 chains tend to have rather regulatory functions [3]. To understand the cellular function of an E3 ligase, it is thus inevitable to characterize its substrates and their respective regulation upon ubiquitination. However, due to the extremely transient nature of the E3 ligase–substrate interaction, conventional interaction-based methods [e.g., immunoprecipitation (IP), yeast two-hybrid] often fail to capture E3 ligase substrate pools completely [4]. On the other hand, powerful techniques such as diGly remnant profiling are elaborate and expensive [4,5]. To tackle this issue, we developed a simple and cost-effective method, which exploits the close proximity of the candidate E3 ligase to both its substrate and the E2~Ub complex. We use a proximity-based labeling approach, which is dependent on ubiquitin (Ub-POD) by tweaking the BioID principle with two essential changes [6]. First, we tagged our candidate E3 ligase with wildtype *E. coli* biotin ligase BirA instead of its highly reactive but promiscuous mutant BirA* (R118G mutation) to restrain its activity to BirA’s acceptor peptide (AP) substrate only. Second, we attached a modified acceptor peptide (-2) AP of BirA to the N-terminus of ubiquitin, rendering it to be selectively biotinylated by BirA WT in a proximity-dependent manner. We then co-transfected these constructs in cells and exposed them to biotin. Upon interaction of the E3 ligase with the E2~ (-2) AP-Ub complex as well as with its substrate, (-2) AP ubiquitin gets biotinylated and transferred to its target, leading to biotin-labeled ubiquitinated substrates. These can be pulled down from cell lysates under denaturing conditions, followed by either digestion of proteins for identification via mass spectrometry (MS) or immunoblotting for substrate validation.

## Materials and reagents

**Biological materials**

1. Target cell line, e.g., human embryonic kidney epithelial cell line 293 (HEK-293) (American Type Culture Collection CRL-1573) (see General note 4)

**Reagents**

1. Dulbecco’s modified Eagle’s medium (DMEM) (Gibco, catalog number: 41965039)

2. Trypsin-EDTA (0.25%), phenol red (Gibco, catalog number: 25200072)

3. Fetal bovine serum (FBS) (Gibco, catalog number: 10270106)

4. Phosphate buffered saline (PBS) (1×) (Gibco, catalog number: 14190169)

5. Opti-MEM (Gibco, catalog number: 31985062)

6. PCR reagents:

a. 5× Phusion HF buffer (New England Biolabs, catalog number: B0518)

b. Phusion HF DNA polymerase (New England Biolabs, catalog number: M0530)

c. dNTP solution mix (New England Biolabs, catalog number: N0447)

d. DMSO (New England Biolabs, catalog number: B0515)

e. Nuclease-free water (New England Biolabs, catalog number: B1500)

7. Template plasmids/cDNAs of the candidate E3 ligases

8. BirA vectors [Addgene, catalog numbers: 232584 (Empty BirA), 232586 (for tagging BirA at the N terminus of the ligase; there is a GSGS linker between BirA and the ligase), 232587 (for tagging BirA at the C terminus of the ligase), and 232588 (for tagging BirA at the C terminus of the ligase; there is a GSGS linker between BirA and the ligase)] and Avi-tagged Ub constructs [Addgene, catalog numbers: 232577, 232582 (this modified Avi-tagged Ub construct (-2)AP-Ub has been used to describe the protocol and also has been used in the original publication), and 232583] (plasmids can be obtained from the following source: https://www.addgene.org/Sagar_Bhogaraju/)

9. Transfection reagents, e.g., polyethyleneimine (PEI) (Polysciences, catalog number: 23966-1), Lipofectamine 2000 (Invitrogen, catalog number: 11668027), or Lipofectamine 3000 (Invitrogen, catalog number: L3000008)

*Note: For optimal results, the transfection reagent should be selected based on the transfection efficiency of the specific cell line of interest.*

10. Biotin (Sigma-Aldrich, catalog number: B4639)

11. MG132 (Calbiochem, catalog number: 474787)

12. Streptavidin agarose (Pierce/Thermo Scientific, catalog number: 20353)

13. Albumin fraction V (BSA) (Carl Roth, catalog number: 8076.3)

14. Benzonase^®^ nuclease (Millipore, catalog number: E1014)

15. cOmplete^TM^, mini protease inhibitor cocktail (Roche, catalog number: 11836153001)

16. Dithiothreitol (DTT) (MP Biomedical, catalog number: 856126)

17. *N*-ethylmaleimide (NEM) (Pierce/Thermo Scientific, catalog number: 23030)

18. Tris base [tris(hydroxymethyl)aminomethane] (Euromedex, catalog number: 200923A)

19. Glycine (Carl Roth, catalog number: 56-40-6)

20. Sodium chloride (NaCl) (Euromedex, catalog number: 1112-A)

21. Triton X-100 (Thermo Scientific Alfa Aesar, catalog number: A16046.AE)

22. SDS (Serva, catalog number: 20765)

23. EGTA (Millipore, catalog number: 324626)

24. Magnesium chloride hexahydrate (MgCl_2_·6H_2_O) (Sigma, catalog number: M2670)

25. Tween-20 (Carl Roth, catalog number: 9127.1)

26. 4× Laemmli sample buffer (Bio-Rad, catalog number: 1610747)

27. 2× Laemmli sample buffer (Bio-Rad, catalog number: 161-0737)

*Note: 2-mercaptoethanol should be added freshly before use to a final concentration of 355 mM to both Laemmli sample buffers.*

28. Color pre-stained protein standard, broad range (10–250 kDa) (NEB, catalog number: P7719)

29. Immobilon Crescendo Western HRP substrate (Millipore, catalog number: WBLUR)

30. Poly-D-Lysine (Gibco, catalog number: A3890401)

31. Paraformaldehyde solution, 4% in PBS (Thermo Scientific, catalog number: J61899.AK)

32. ProLong^TM^ diamond antifade mountant with DAPI (Invitrogen, catalog number: P36966)

33. PR-619 [Tocris (Biotechne), catalog number: 4482]

34. USP2 (R&D Systems, catalog number: E504)

35. 2-Mercaptoethanol (Gibco, catalog number: 21985023)

36. Sodium phosphate, dibasic (Na_2_HPO_4_·2H_2_O) (Carl Roth, catalog number: 4984.1)

37. Potassium phosphate, monobasic (KH_2_PO_4_) (Carl Roth, catalog number: 3904.1)

**Antibodies**

1. IRdye streptavidin 680 (Licor, catalog number: 926-32230)

2. IRdye streptavidin 800 (Licor, catalog number: 926-68079)

3. HA-Tag (C29F4) rabbit mAb (Cell Signaling Technology, catalog number: 3724-S)

4. Avi-tag (Genscript, catalog number: A00674)

5. Alpha-Tubulin (anti-mouse) (Bio-Techne Ltd., catalog number: NB100-690SS)

6. Goat anti-mouse IgG (H+L) secondary antibody, HRP (SantaCruz, catalog number: sc-516102)

7. Goat anti-rabbit IgG (H+L) secondary antibody, HRP (SantaCruz, catalog number: sc-2357)

**Solutions**

1. 1 M DTT (see Recipes)

2. 1 mM Biotin (see Recipes)

3. 10× phosphate buffered saline (PBS) (pH 7.4) (see Recipes)

4. 1 M Tris HCl, pH 7.5 (see Recipes)

5. 5 M NaCl (see Recipes)

6. 10% SDS (see Recipes)

7. 100 mM EGTA (see Recipes)

8. 1 M MgCl_2_·6H_2_O (see Recipes)

9. 2 M NEM (see Recipes)

10. 1× Cell Lysis Buffer (CLB) (see Recipes)

11. IP Wash buffer 1 (see Recipes)

12. IP Wash buffer 2 (see Recipes)

13. IP Wash buffer 3 (see Recipes)

14. 10× running buffer (see Recipes)

15. 10× transfer buffer (see Recipes)

16. 1× PBST (see Recipes)

17. Blocking buffer (see Recipes)

18. Antibody dilution buffer for streptavidin immunoblotting (see Recipes)

19. Blocking and permeabilization buffer for streptavidin immunofluorescence microscopy (see Recipes)

20. Antibody dilution buffer/wash buffer for streptavidin immunofluorescence microscopy (see Recipes)

**Recipes**

**1. 1 M DTT**

Dissolve 1.5425 g of DTT in 8 mL of distilled water. Adjust volume to 10 mL. Filter through a 0.22 μm filter. Aliquot and store in the dark at -20 °C.

**2. 1 mM Biotin**

Dissolve 12.2155 mg of biotin powder in 50 mL of serum-free DMEM. Vortex. Filter through a 0.22 μm filter. Make aliquots to prevent freeze-thawing.

**3. 10× phosphate buffered saline (PBS) (pH 7.4)**

**Table:** 

Reagent	Final concentration	Quantity or Volume
NaCl	1.37 M	80 g
KCl	27 mM	2 g
Na_2_HPO_4_⋅2H_2_O	100 mM	17.8 g
KH_2_PO_4_	18 mM	2.4 g
Distilled water	n/a	Up to 1 L

Dissolve all the reagents in 800 mL of distilled water. Adjust the pH to 7.4. Fill the volume up to 1 L with distilled water. Filter through a 0.22 μm filter and store at room temperature (RT) or 4 °C.

**4. 1 M Tris HCl (pH 7.5)**

Dissolve 121.14 g of Tris base in 800 mL of distilled water. Adjust to the desired pH with concentrated HCl. Mix and add distilled water to 1 L. Sterilize by autoclaving and store at RT.

**5. 5 M NaCl**

Dissolve 292.2 g of NaCl powder in 1 L of distilled water. Sterilize by autoclaving and store at RT.

**6. 10% SDS**

Dissolve 10 g of SDS in 80 mL of distilled water. Fill the volume up to 100 mL. Dissolve the solution on a magnetic stirrer. If needed, heat the solution slightly to dissolve the powder. Store at RT.

**7. 100 mM EGTA**

Dissolve 3.804 g of EGTA in 10 mL of 1.7 M Tris solution. Add 80 mL of distilled water and adjust pH to 7.0 with 1.7 M Tris solution. Bring the final volume to 100 mL with distilled water. The solution may be stored at RT after sterilizing by autoclaving.

**8. 1 M MgCl_2_·6H_2_O**

Dissolve 20.33 g of MgCl_2_⋅6H_2_O in 100 mL of distilled H_2_O. This solution is extremely hygroscopic. Do not store open bottles for longer periods. Sterilize by autoclaving and store at RT.

**9. 2 M NEM**

Dissolve 12.513 g of NEM in 50 mL of isopropanol. Aliquot and store at -20 °C.

**10. 1× cell lysis buffer (CLB)**

**Table:** 

Reagent	Final concentration	Volume
1 M Tris-HCl, pH 7.5	50 mM	2.5 mL
5 M NaCl	150 mM	1.5 mL
Triton X-100	1%	500 μL
10% SDS	0.1%	500 μL
100 mM EGTA	1 mM	500 μL
1 M MgCl_2_⋅6H_2_O	1.5 mM	75 μL
Benzonase	250 U/mL	50 μL
2 M NEM	50 mM	1.25 mL
Protease inhibitor cocktail	n/a	1 tablet/50 mL
MilliQ water	n/a	up to 50 mL

Store at -20 °C.

**11. IP wash buffer 1**

**Table:** 

Reagent	Final concentration	Volume
1 M Tris-HCl, pH 7.5	50 mM	2.5 mL
5 M NaCl	500 mM	5 mL
Triton X-100	1%	500 μL
10% SDS	1%	5 mL
1 M DTT	10 mM	500 μL
100 mM EGTA	1 mM	500 μL
2 M NEM	50 mM	1.25 mL
Protease inhibitor cocktail	n/a	1 tablet/50 mL
MilliQ water	n/a	up to 50 mL

Add SDS and DTT freshly before use.

**12. IP wash buffer 2**

**Table:** 

Reagent	Final concentration	Volume
1 M Tris-HCl, pH 7.5	50 mM	2.5 mL
5 M NaCl	150 mM	1.5 mL
Triton X-100	1%	500 μL
10% SDS	0.1%	500 μL
1 M DTT	10 mM	500 μL
100 mM EGTA	1 mM	500 μL
2 M NEM	50 mM	1.25 mL
Protease inhibitor cocktail	n/a	1 tablet/50 mL
MilliQ water	n/a	up to 50 mL

Add DTT freshly before use.

**13. IP Wash buffer 3**

**Table:** 

Reagent	Final concentration	Volume
1 M Tris-HCl, pH 7.5	50 mM	2.5 mL
5 M NaCl	150 mM	1.5 mL
2 M NEM	50 mM	1.25 mL
Protease inhibitor cocktail	n/a	1 tablet/50 mL
MilliQ water	n/a	up to 50 mL

**14. 10**× **running buffer**

**Table:** 

Reagent	Final concentration	Quantity
Tris base	250 mM	30.285 g
Glycine	1.92 M	144.4 g
SDS	1% (w/v)	10 g
Distilled water	n/a	up to 1 L

Store at RT and dilute to 1× before use.

**15. 10**× **transfer buffer**

**Table:** 

Reagent	Final concentration	Quantity
Tris base	250 mM	30.285 g
Glycine	1.92 M	144.4 g
Distilled water	n/a	Up to 1 L

Before use, make 1× transfer buffer by diluting 10× transfer buffer and adding 20% methanol.

**16. 1**× **PBST**

**Table:** 

Reagent	Final concentration	Volume
10× PBS	1×	100 mL
Tween 20	0.2%	2 mL
Distilled water	n/a	up to 1 L

**17. Blocking buffer**

**Table:** 

Reagent	Final concentration	Quantity
BSA	5%	5 g
1× PBS	n/a	100 mL

Store at 4 °C.

**18. Antibody dilution buffer for streptavidin immunoblotting**

**Table:** 

Reagent	Final concentration	Quantity
BSA	5%	0.5 g
1× PBS	n/a	10 mL
Tween-20	0.2%	20 μL
10 % SDS	0.1%	100 μL

Add SDS and Tween-20 freshly before use.

**19. Blocking and permeabilization buffer for streptavidin immunofluorescence microscopy**

**Table:** 

Reagent	Final concentration	Quantity
BSA	5%	5 g
Triton X-100	0.3%	300 μL
1× PBS	n/a	up to 100 mL

Store at 4 °C.

**20. Antibody dilution buffer/wash buffer for streptavidin immunofluorescence microscopy**

**Table:** 

Reagent	Final concentration	Quantity
BSA	3%	15 g
Triton X-100	0.1%	500 μL
1× PBS	n/a	up to 500 mL

Store at 4 °C.

**Laboratory supplies**

1. 10 cm cell culture dishes (Corning, catalog number: 430167)

2. 6-well cell culture plates (Corning, catalog number: 353046)

3. Cell lifter/scraper (Biologix, catalog number: 70-2180)

4. Microtubes 0.5 mL (Treff, catalog number: 61-9708-73), 1.5 mL (Eppendorf, catalog number: 0030120086), and 2 mL (Eppendorf, catalog number: 0030120094)

5. 0.2 mL PCR tubes (StarLab, catalog number: I1402-8200)

6. Falcon centrifuge tubes, 15 mL (Corning, Falcon, catalog number: 352096) and 50 mL (Corning, Falcon, catalog number: 352070)

7. 4%–20% Mini-PROTEAN^®^ TGX precast protein gels, 10-well (Bio-Rad, catalog number: 4561093)

8. Microscope slides (Epredia^TM^, catalog number: 17244884)

9. Cover glass (Fisherbrand, catalog number: 12333138)

## Equipment

1. SDS-PAGE electrophoresis apparatus and chambers (Bio-Rad, catalog number: 1658004)

2. SDS-PAGE transfer apparatus (Bio-Rad, catalog number: 1703935)

3. Thermoblock (Thermo Fisher Scientific, catalog numbers: 13687717, 13687718)

4. Thermomixer (Boekel Scientific, catalog numbers: 270811, 270812)

5. Sonicator (Branson 450 Digital Sonifier; Bioruptor^®^ Pico sonication device, catalog number: B01080010)

6. Cell culture incubator (Heracell^TM^ 150i CO_2_ Incubator, 150 L, Thermo Scientific^TM^, catalog number: 51032720)

7. Gentle rotating mixer for Eppendorf tubes (end-over-end rotation) (VWR, catalog number: 10136-084)

8. Centrifuge for 15 and 50 mL tubes (Eppendorf, model: 5804)

9. Centrifuge for 1.5 mL reaction tubes with cooling function (Eppendorf, model: 5427 R)

10. Micro-pipettes (Gilson, catalog number: F167300)

11. NanoDrop 2000 UV-Vis spectrophotometer (Thermo Fisher Scientific, Thermo Scientific^TM^, model: NanoDrop^TM^ 2000, catalog number: ND-2000)

12. PCR machine (Bio-Rad, model: T100 THERMAL CYCLER, catalog number: 1861096)

13. -80 °C freezer

14. -20 °C freezer

15. Refrigerator (4 °C)

16. Chemidoc MP imaging system (Bio-Rad, catalog number: 12003154)

17. Confocal microscope with 63× oil-immersion objective (Leica, model: TCS SP5)

## Software and datasets

1. Image Lab software v5.2.1 (Bio-Rad)

2. Excel (Microsoft)

3. GraphPad Prism 9 (GraphPad)

4. ImageJ (Version 1.49s, National Institutes of Health)

5. Adobe Illustrator CS6 (Version 16.0.3)

## Procedure

**A. Cloning of E3 ligase-BirA fusion constructs and (-2) AP-Ub construct**

1. Create E3 ligase-BirA fusion constructs by tagging BirA at either end of the candidate E3 ligase.

**Figure 1.**
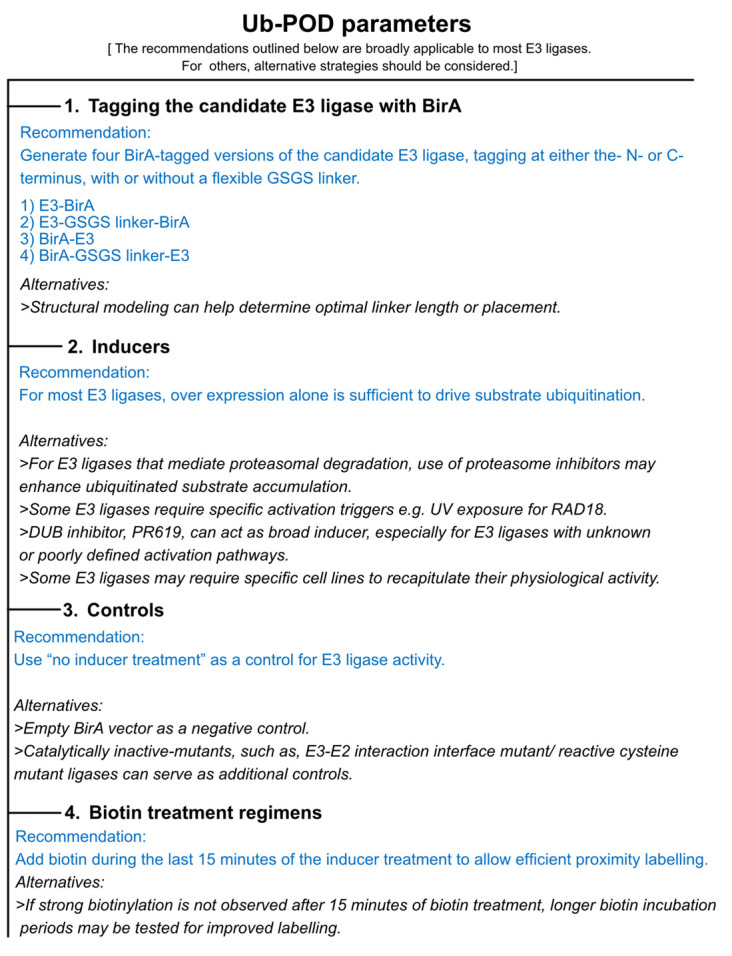
Key experimental parameters to consider when performing ubiquitin-specific proximity-dependent labeling (Ub-POD) analysis for a candidate ubiquitin ligase

*Note: Trial and error with various E3 ligases in our laboratories suggests that, at least for some E3 ligases, Ub-POD is sensitive to the location of the BirA tag with respect to the catalytic domain in the E3 ligase. When structural information is available, it is best to make a structure-based decision regarding the placement of the BirA tag so as not to interfere with its binding to, e.g., the Cullin scaffold. When there is no structural information available, we recommend creating four different BirA-tagged versions, where the ligase is tagged at the N- and C-termini with or without a linker (see the list of BirA vectors in the Materials and reagents section above) (Figure 1). An optimally placed BirA tag would result in a “standout” biotinylation of cellular proteins (potential substrates) when compared to the other constructs with different BirA tag placements, as well as when compared to BirA alone (also, see General notes 1 and 2).*

**Figure 2.**
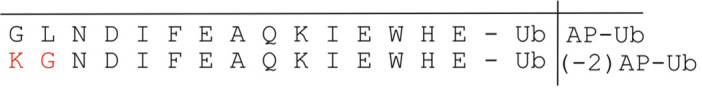
Sequence comparison of AP-Ub and (-2) AP-Ub variants

2. Create (-2) AP-Ub construct by tagging a modified acceptor peptide (see Figure 2) at the N terminus of Ub (also, see General note 3).

*Note: AP has a high intrinsic affinity for BirA, giving rise to nonspecific background biotinylation signals. Thus, to achieve specific AP biotinylation strictly depending on proximity to the BirA-labeled E3 ligase, we changed two N-terminal amino acids of AP. This variant of the AP tag is derived from Fernández-Suárez et al. [7].*

**B. Optimizing the E3 ligase–BirA fusion constructs and experimental conditions by streptavidin immunoblotting**

1. Transient transfection of E3 ligase–BirA fusion constructs and (-2) AP-Ub

a. Day 1: Seed HEK-293 cells at a density of 1 × 10^5^ cells in 2 mL of culture medium per well of a 6-well plate to achieve ~70% confluency the following day. Incubate the cells overnight at 37 °C with 5% CO_2_ in a cell culture incubator (see General note 4).

b. Day 2: For each well to be transfected, dilute the plasmid constructs in Opti-MEM as outlined in Table 1.

**Table 1.** Plasmid constructs and quantities for transfection

Plasmid constructs	Quantities	Opti-MEM
Empty BirA + (-2) AP-Ub	1.5 μg each	250 μL
BirA-E3 fusion construct + (-2) AP-Ub	1.5 μg each	250 μL
E3-BirA fusion construct + (-2) AP- Ub	1.5 μg each	250 μL

c. Dilute PEI in 250 μL of Opti-MEM medium (~3 μg of PEI per 1 μg DNA) (per well of a 6-well plate).

d. Add the diluted PEI to the diluted DNA in each tube and mix gently.

e. Incubate the mixture at room temperature for 15–20 min.

f. Add the transfection mix dropwise to the cells.

g. Incubate the cells for 16–24 h.

2. Proximity biotinylation and/or inducer treatment: different biotin treatment regimens should be tested for each ligase. Biotin treatment can range from 15 min to 24 h (Figure 1).

**Regime 1:**

a. Discard the transfection medium.

b. Add 50–100 μM biotin in complete medium to the transfected cells.

c. If required, add specific inducers to trigger ubiquitination of target proteins by the ligase of interest (see General note 5 and Figure 1).

d. Incubate for 15 min to 6 h, depending on the ligase of interest (see General notes 5 and 6).

Or

**Regime 2:**

e. Add 50–100 μM biotin directly to the transfection medium during transfection.

f. Incubate for 16 h.

g. Discard the transfection medium.

h. Add 50–100 μM biotin and specific inducers to the cells.

i. Incubate for 15 min to 6 h, depending on the ligase of interest (see General note 6).

3. Cell lysate preparation

a. Wash the cells once with 1 mL of cold (4 °C) 1× PBS. Discard the PBS.

b. Add 500 μL of cold (4 °C) 1× PBS to each well.

c. Harvest the cells using a cell scraper and transfer to 1.5 mL tubes.

d. Centrifuge the samples at 500× *g* for 5 min at 4 °C.

e. Discard the supernatants and proceed to lysis. Alternatively, snap-freeze the pellets in liquid nitrogen and store at -80 °C until further processing.

f. Add 100 μL of 1× CLB to each pellet.

g. Dissolve the pellet in 1× CLB by pipetting (see General note 7).

h. Sonicate the lysate in a water bath for 5 min at 4 °C.

i. Lyse the cells on ice for 30 min, mixing the lysate by pipetting every 10 min.

j. Centrifuge the lysates at 14,000× *g* for 10 min at 4 °C to remove debris.

k. Transfer the supernatants to fresh tubes and keep on ice.

l. Determine protein concentration using a BCA assay.

m. Prepare samples with 4× Laemmli sample buffer and heat at 95 °C for 5–10 min.

4. SDS-polyacrylamide gel electrophoresis (PAGE) and western blotting

a. Load equal amounts of protein (10–20 μg) per sample into 4%–20% precast gels and perform SDS-PAGE.

b. After electrophoresis, transfer the fractionated proteins to PVDF membranes using a standard protocol.

*Note: For streptavidin immunoblotting, low-fluorescence PVDF membranes are recommended.*

c. Block the membranes in blocking buffer for 1 h at RT.

d. For streptavidin detection, incubate the membrane with fluorescent streptavidin conjugate (IRdye streptavidin 680/IRdye streptavidin 800) (1:5,000 dilution) in antibody dilution buffer for 45–60 min. From this step onward, protect the membrane from light (see General note 8).

e. Wash the membrane three times for 5 min each with wash buffer, followed by one wash with 1× PBS.

f. Scan the membrane using a Chemidoc MP imaging system (fluorescence scanner).

g. Re-probe the membrane with anti-HA-tag and anti-Ub/anti-Avi tag antibodies (for checking transfection efficiency) and anti-tubulin antibody (for loading control).

h. Select the E3-BirA/BirA-E3 fusion construct and biotin/inducer treatment condition that demonstrates optimal (stronger) streptavidin signal relative to similarly treated BirA control for subsequent experiments.

**C. Ub-POD for confocal imaging**

1. Seeding HEK-293 cells on coated coverslips

a. Disinfect the 22 × 22 mm coverslips with 70% ethanol and place them into 6-well plates.

b. Dilute the Poly-D-Lysine solution in sterile 1× PBS to prepare a 50 μg/mL working solution.

c. Add 1.5 mL of Poly-D-Lysine solution to each well to coat the surface of the coverslips in the 6-well plate.

d. Incubate the plates at room temperature for 1 h.

e. Remove the Poly-D-Lysine solution.

f. Rinse the wells three times with sterile 1× PBS (2 mL/well for each wash),

g. Remove the final wash and leave the plate in a laminar hood to dry for at least 2 h.

h. Seed HEK-293 cells onto the coated coverslips to ensure they reach at least 70% confluency the following day.

2. Transient transfection of optimal E3-BirA fusion construct, (-2) AP-Ub, and biotin and/or inducer treatment

a. Transfect E3-ligase BirA fusion construct and (-2) AP-Ub as mentioned in step B1. Include wells co-transfected with empty BirA and (-2) AP-Ub as controls.

b. Incubate the cells for 16–24 h.

c. Treat the cells with biotin and specific inducers as optimized in step B2.

d. Aspirate the media from each well and wash the cells at least three times with 500 μL of 1× PBS to remove excess biotin.

3. Fixation

a. Fix the cells by adding 500 μL of 4% paraformaldehyde solution to each well. Incubate for 15–20 min at room temperature.

b. After incubation, discard the paraformaldehyde solution in an appropriate hazardous liquid waste container and wash the cells three times with 500 μL of 1× PBS to remove any residual fixative.

4. Permeabilization and blocking

a. Permeabilize and block the cells simultaneously by incubating them with 500 μL of permeabilization and blocking buffer/well for 1–3 h at room temperature.

5. Primary antibody staining

a. Aspirate the permeabilization and blocking buffer from the wells.

b. Wash the cells once with 1× PBS.

c. Dilute the anti-HA-tag primary antibody in the antibody dilution buffer according to the manufacturer’s recommendations.

*Note: Primarily, anti-HA-tag staining should be performed to assess the transfection efficiency of the empty BirA/E3 ligase-BirA fusion construct. Alternatively, other primary antibodies can be used to detect the localization/downstream function of the ligase of interest.*

d. Incubate the coverslips with the primary antibody overnight at 4 °C in a moist chamber.

6. Streptavidin and secondary antibody staining

a. Aspirate the primary antibody solution.

b. Wash the coverslips three times with 500 μL of antibody dilution buffer, followed by two washes with 1× PBS.

c. Incubate the coverslips with streptavidin-conjugated to IRdye 680 (1:200) and Alexa Fluor 488–conjugated anti-rabbit secondary antibody (1:200) (for anti-rabbit anti-HA-Tag primary antibody) in antibody dilution buffer for 1 h in the dark in a humidified 37 °C incubator.

d. Wash the cells five times with wash buffer (see Recipe 20) and once with 1× PBS.

*Note: When selecting fluorophores for secondary antibody staining, ensure compatibility with the excitation/emission spectra of streptavidin-conjugated IRdye 680 and DAPI to allow co-staining.*

7. Mounting coverslips and confocal imaging

a. Add 1 drop of antifade mounting medium, containing the nuclear stain DAPI, onto a cleaned microscope slide.

b. Carefully remove the cover glasses from the wells and gently blot the edges to remove excess liquid.

c. Place the coverslip onto the slide, ensuring no air bubbles form.

d. Wipe off any excess mounting medium from the edges and store the slides in the dark at room temperature.

e. Perform confocal imaging using a confocal laser scanning microscope with 63× oil immersion objective, ensuring consistent parameters are maintained across all samples.

**D. Ub-POD for proteomics studies**

For proteomics, all steps must be performed under keratin-free conditions. Ensure that all materials and reagents used are as keratin-free as possible.

1. Transient transfection of the optimal E3-BirA fusion construct and (-2) AP-Ub

a. Day 1: Seeding

Seed four 10 cm tissue culture dishes per condition with 8 × 10^5^ cells in 10 mL of complete medium per plate. Incubate overnight at 37 °C with 5% CO_2_ in a cell culture incubator.

b. Day 2: Transfection

For each plate, dilute the plasmid constructs in Opti-MEM as shown in Table 2.

**Table 2.** Plasmid combinations and quantities for transient transfection

Condition	Plasmid constructs	Opti-MEM
Empty BirA + (-2) AP-Ub	6 μg each	500 μL
E3-BirA + (-2) AP- Ub	6 μg each	500 μL

c. Dilute PEI in 500 μL of Opti-MEM (~3 μg of PEI per 1 μg of DNA) (per 10 cm dish).

d. Incubate each master mix for at least 5 min at RT.

e. Mix diluted PEI with diluted DNA and incubate for 15–20 min at RT.

f. Add 1 mL of the transfection mixture dropwise to each plate. Incubate for 16–24 h at 37 °C with 5% CO_2_.

2. Induction and proximity-labeling

a. The day after transfection, remove the medium and wash the cells with 5 mL of 1× PBS.

b. Add 10 mL of complete medium to each plate supplemented with 50–100 μM of biotin and the appropriate inducer.

c. Incubate the cells for 15 min to 6 h at 37 °C with 5% CO_2_.

3. Harvesting the cells

a. Wash the cells three times with ice-cold 1× PBS. Add 1.5 mL of 1× PBS to each plate and harvest the cells using a cell scraper.

b. Transfer the harvested cells corresponding to one condition to a 15 mL tube and pellet by centrifugation at 500× *g* for 5 min at 4 °C.

c. Remove the supernatants and proceed to streptavidin IP.

d. Alternatively, snap-freeze the pellets in liquid nitrogen and store at -80 °C until further processing.

4. Preparation of cell lysates

a. Resuspend the cell pellets in 1 mL of 1× CLB (see General note 7).

b. Sonicate the samples in a cold (4 °C) water bath.

c. Further lyse the cells on ice for 30 min, vortexing/pipetting every 10 min.

d. Centrifuge the lysates at 14,000× *g* for 10 min at 4 °C.

e. Measure protein concentration using a BCA assay. Use 1–2 mg of protein for streptavidin pulldown. Keep 2%–5% lysate as input.

*Note: For Ub-POD proteomics studies, we recommend using a minimum of 1 mg of protein. The optimal protein concentration should be determined based on the results of streptavidin immunoblotting. One approach to assess whether the protein amount is sufficient for identifying substrates is to evaluate the expression of a known substrate of the candidate E3 ligase by streptavidin IP followed by immunoblotting with a specific antibody.*

5. Streptavidin pulldown

a. Transfer 40–60 μL of streptavidin-coupled agarose beads suspension to a 1.5 mL tube for each condition (see General note 9).

b. Wash the beads twice with 500 μL of 1× CLB.

• Gently mix the beads with CLB.

• Centrifuge at 500× *g* for 2 min (see General note 10).

• Discard the supernatant.

c. Add 500 μL of clarified lysate (containing at least 1–2 mg protein) to the washed beads. Ensure a minimum volume of 500 μL for proper mixing.

d. Incubate at 4 °C for at least 2 h on a rotating wheel (see General note 11).

e. After incubation, transfer the supernatant (flowthrough) to a 1.5 mL tube for analysis.

f. Resuspend the beads in each tube with 500 μL of wash buffer 1.

g. Wash the beads sequentially as follows:

• Gently add wash buffer to the beads.

• Incubate the beads for 5 min on a rotation wheel at 4 °C.

• Centrifuge at 500× *g* for 2 min.

• Keep supernatant in a separate tube for analysis (Table 3).

**Table 3.** Wash buffers and parameters for streptavidin pulldown

Wash buffer	Number of washes	Volume (per wash)
IP Wash buffer 1	2	500 μL
IP Wash buffer 2	2	500 μL
IP Wash buffer 3	2	500 μL

h. Resuspend the beads in 50 μL of 2× Laemmli buffer.

i. Boil the samples at 95 °C for 15 min.

ii. Centrifuge at 16,000× *g* for 5 min at RT. Transfer the eluates to fresh tubes. Keep 10% of the elute for checking the efficiency of the IP and store the rest at -20 °C until further processing.

*Note: If 2× Laemmli buffer is used for elution, samples should be cleaned using the SP3 protocol (Hughes et al. [8]) prior to mass spectrometry analysis. Alternatively, 3 M urea buffer can be used for elution.*

6. SDS-PAGE and western blotting

Prior to mass spectrometry analysis, it is recommended to assess the success of the biotinylation and pulldown by SDS-PAGE and immunoblot.

a. Load the following samples on an SDS polyacrylamide gel.

• Input: 15–30 μg proteins/well

• IP reaction: 10 μL/well

b. If performing the pulldown for the first time, prepare PAGE samples for flowthrough and washes (wash 1–4) and load on an SDS polyacrylamide gel.

• Mix 20 μL of sample (flowthrough, wash 1–4) with 5 μL of 4× SDS loading buffer.

c. Perform electrophoresis and western blotting as described in step B4.

7. Proteomics and data analysis

Proceed with mass spectrometry analysis to identify the biotinylated-ubiquitinated proteins. For quantitative comparisons, either tandem mass tags (TMT) or label-free quantification methods can be applied. The data can be processed using proteomics software platforms such as MaxQuant or MSFragger. Statistical filtering should be considered to ensure that only high-confidence hits (e.g., FDR < 0.05) are retained.

## Validation of protocol

This protocol has been used and validated in:

• Mukhopadhyay et al. [6]. A ubiquitin-specific, proximity-based labeling approach for the identification of ubiquitin ligase substrates. *Sci Adv*.

Controls that were used to validate the protocol:

• Successful biotinylation for BirA-E3 and (-2) AP-Ub expressing cells was checked via immunoblotting (Figures 3A and 4A from [6]). This biotinylation was shown to be ubiquitin-specific, since conjugation-deficient (-2) AP-Ub^ΔGG ^mutant abolished protein biotinylation completely (Figures 2D, S5C, and 4D from [6]).

• MS experiments were conducted in triplicate or quadruplicate per condition, and statistical testing was performed (Figures 2B, 3C, and 4B from [6]).

• Identified substrates via MS were validated via denaturing streptavidin pulldown and immunoblotting. The substrates were efficiently pulled down, and ubiquitylation could be visualized by a size shift to higher molecular weight (Figures 2D, 3E, and 4D from [6]). The pulldown of ubiquitinated species was furthermore validated by treating pulled-down proteins with deubiquitinase USP2, resulting in loss of the substrates from the IP fraction (Figures S2C, S5D, and S6G from [6]).

## General notes and troubleshooting

**General notes**

1. When designing the E3 ligase-BirA fusion constructs, it is essential to ensure that the fusion of BirA does not disrupt the functionality of the E3 ligase. Testing both E3 ligase-BirA and BirA-E3 ligase constructs is recommended to evaluate the effect of the BirA tag's position on the ligase’s activity.

2. Incorporating a GSGS linker between the ligase and the BirA tag may enhance substrate identification by Ub-POD. The linker length may need to be optimized for each candidate ligase and could be case-specific.

3. We have successfully used (-2) AP-Ub to optimize Ub-POD and performed experiments using this Avi-tagged variant [6]. However, alternatives such as AP (-3) Ub [9,10] or other AP variants described in [7] can also be utilized in place of (-2) AP-Ub.

4. Any cell line can be used for Ub-POD. However, the transfection efficiency of the cell line should be at least 50%–60% for transient transfection.

5. For certain E3 ligases, overexpression alone can trigger ligase-mediated substrate ubiquitination. For E3 ligases with known activation triggers, these should be applied. For novel E3 ligases where the activation trigger is not known, a proteasome inhibitor (e.g., MG132) or a deubiquitinase inhibitor can be used to preserve ubiquitination.

6. The timing of biotin addition and duration of biotin exposure are highly case-specific and should be optimized for each ligase. Biotin treatments ranging from 15 min to 24 h have been tested and yielded positive results [6,9,10].

7. RIPA buffer can be used as an alternative to the recommended cell lysis buffer.

8. This protocol uses a fluorescent-based detection, but a luminescence-based detection using HRP-coupled streptavidin/biotin antibodies works equally well for detecting biotinylated proteins.

9. Streptavidin-coupled magnetic beads should perform equally well for the pulldown, though the protocol may need to be adjusted accordingly.

10. When centrifuging the beads, it is recommended to use a “soft mode” on the centrifuge to avoid damaging the beads.

11. The streptavidin-coupled beads can be incubated with the clarified lysates from 2 h to overnight. The optimal incubation time should be determined based on proteomics results.

12. The use of proper negative controls is crucial for minimizing background signals. Negative controls may include a) non-fused BirA, b) ligase catalytic inactive mutant fused to BirA, c) conjugation deficient (-2) AP-Ub^ΔGG^, and d) comparison of mock vs. E3 activating conditions.

13. Ubiquitination is a reversible process, and after biotinylation and transfer to its substrate, the ubiquitin moiety could be cleaved off by deubiquitinases. This could lead to the recycling of biotin-labeled ubiquitin in BirA-E3 ligase-independent reactions. To mitigate this issue, the addition of deubiquitinase inhibitors (e.g., PR-619) during the biotinylation reaction, cell lysis, and pulldown is recommended. Ubiquitin recycling might be particularly problematic for Ub-POD experiments that require extended biotin exposure and ubiquitin events, which are rapidly regulated through DUBs.

14. Ub-POD has been successfully tested with RING E3 ligases (the largest family of E3 ligases with ~600 members) and U-box E3 ligases but can also be applied to other E3 families, as demonstrated in other studies using similar approaches [6].

15. Ub-POD can, in principle, be adapted to identify substrates of other ubiquitin-like molecules (UBLs).

**Troubleshooting**

**Problem 1:** Fusion constructs do not express correctly or show weak signal.

Ensure proper alignment of the BirA tag with the ligase's functional domain. The position of the BirA tag relative to the ligase’s catalytic site may need optimization.

**Problem 2:** Successfully transfected cells show no biotinylation upon biotin exposure.

• Ensure that the biotin is freshly prepared.

• One reason could be that the biotin exposure is too short or too long. Do a biotin time course to find the best treatment time point.

• Another reason could be that the E3 ligase shows no activity under steady-state conditions and needs to be stimulated by a certain perturbation. Test biotin exposure in combination with E3 ligase-activating stimulants.

• Further, ubiquitylation events that result in proteasomal degradation of substrates might need additional treatment with proteasome inhibitors (e.g., MG-132) to accumulate substrates.

• If none of the mentioned approaches help, it might be beneficial to try both C- and N-terminal tagged variants of BirA-E3 fusions.

• Also, introducing a linker sequence between E3 and BirA can introduce additional structural flexibility and thus better orientation of BirA to the E2~(-2) AP-Ub complex.

**Problem 3:** Biotinylation of cells expressing a catalytically inactive mutant E3-BirA is observed.

Many E3 ligases act as dimers. Thus, even catalytic dead mutants might still be partially functional when dimerizing with endogenous wild-type E3. Thus, working in KD or KO settings is advantageous.

**Problem 4:** Low streptavidin signal in western blot.

Ensure membrane blocking is done for a sufficient time, and use low-fluorescence PVDF membranes for better detection.

**Problem 5:** Weak pulldown signal.

If pulldown efficiency is low, increase incubation time with beads or adjust bead-to-lysate ratio. If necessary, try using a more concentrated lysate or increasing bead volume.
